# Novel PD-L1-Targeted Phenyl-Pyrazolone Derivatives with Antioxidant Properties

**DOI:** 10.3390/molecules28083491

**Published:** 2023-04-15

**Authors:** Romain Regnault, Frédérique Klupsch, Hassiba El-Bouazzati, Romain Magnez, Raphaël Le Biannic, Natascha Leleu-Chavain, Hania Ahouari, Hervé Vezin, Régis Millet, Jean-François Goossens, Xavier Thuru, Christian Bailly

**Affiliations:** 1 ULR 7365—GRITA—Groupe de Recherche sur les formes Injectables et les Technologies Associées, CHU Lille, University Lille, F-59000 Lille, France; romain.regnault@univ-lille.fr (R.R.); jean-francois.goossens@univ-lille.fr (J.-F.G.); 2 U1286—INFINITE—Institute for Translational Research in Inflammation, ICPAL, Inserm, University Lille, F-59000 Lille, Franceregis.millet@univ-lille.fr (R.M.); 3 UMR9020—UMR1277—Canther—Cancer Heterogeneity, Plasticity and Resistance to Therapies, Inserm, CNRS, CHU Lille, University Lille, F-59000 Lille, France; 4 LASIRE Laboratoire Avancé de Spectroscopie pour les Intéractions la Réactivité et l’Environnement, F-59655 Villeneuve d’Ascq, France; 5 FR 2638—IMEC—Institut Michel-Eugène Chevreul, University Lille, F-59655 Lille, France; 6 Oncowitan, Scientific Consulting Office, Wasquehal, F-59290 Lille, France

**Keywords:** aldehyde reactivity, antioxidant, cancer, immune checkpoint, PD-L1 binding, pyrazolone

## Abstract

Orally-active anticancer small molecules targeting the PD-1/PD-L1 immune checkpoint are actively searched. Phenyl-pyrazolone derivatives with a high affinity for PD-L1 have been designed and characterized. In addition, the phenyl-pyrazolone unit acts as a scavenger of oxygen free radicals, providing antioxidant effects. The mechanism is known for the drug edaravone (**1**) which is also an aldehyde-reactive molecule. The present study reports the synthesis and functional characterization of new molecules (**2**–**5**) with an improved anti-PD-L1 activity. The leading fluorinated molecule **5** emerges as a potent checkpoint inhibitor, avidly binding to PD-L1, inducing its dimerization, blocking PD-1/PD-L1 signaling mediated by phosphatase SHP-2 and reactivating the proliferation of CTLL-2 cells in the presence of PD-L1. In parallel, the compound maintains a significant antioxidant activity, characterized using electron paramagnetic resonance (EPR)-based free radical scavenging assays with the probes DPPH and DMPO. The aldehyde reactivity of the molecules was investigated using 4-hydroxynonenal (4-HNE), which is a major lipid peroxidation product. The formation of drug-HNE adducts, monitored by high resolution mass spectrometry (HRMS), was clearly identified and compared for each compound. The study leads to the selection of compound **5** and the dichlorophenyl-pyrazolone unit as a scaffold for the design of small molecule PD-L1 inhibitors endowed with antioxidant properties.

## 1. Introduction

Despite the progress of immunotherapy, the quest for new anticancer drugs and treatments remains a priority for most patients and their clinicians in many countries. Monoclonal antibodies targeting immune checkpoints, such as the master checkpoint PD-1/PD-L1, have contributed to improve the outcome of many patients with solid tumors, including melanoma, non-small cell lung cancer, and renal cancer. However, for other malignancies, the benefit of these immunotherapies has remained limited. Novel therapeutic options are needed. One option is the design of orally-active small molecules targeting immune checkpoints, to propose novel combinations with chemotherapy and/or targeted therapies [1].

In this context, different drug scaffolds have been proposed to target the ligand protein PD-L1 (Programmed Death-Ligand 1) so as to block its interaction with the receptor PD-1 (Programmed cell Death-1). The first PD-L1-targeted inhibitors were proposed in 2017, based on the discovery of the mode of binding to PD-L1 dimer of biphenyl-type compounds from Bristol-Myers Squibb [2,3]. Over the past 6 years, different chemical series of small molecules targeting PD-L1 have been proposed, such as biphenyl-based compounds [4,5], benzamide derivatives [6], benzo-isothiazoles [7], phthalimide derivatives [8] and many others. New molecules are regularly proposed, notably with the support of molecular modeling approaches to guide drug design [9,10,11]. There are several PD1/PD-L1 small molecule inhibitors in preclinical studies but a very few compounds in early-stage clinical trials at present such as the drug candidates INCB-086550 (Incyte Corp., Wilmington, DE, USA) and IMMH-010 (Tianjin Chasesun Pharmaceutical Co., Tianjin, China) for examples [12,13]. It is a challenge to develop an orally-active PD-L1 inhibitor for cancer therapy.

**Figure 1:**
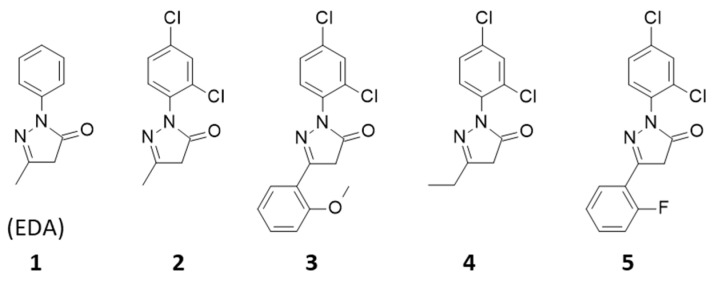
Structures of compounds **1**–**5**.

In this context, we have previously proposed an innovative series of PD-L1 targeted small molecules bearing a phenyl-pyrazolone scaffold, designated PyrDLones, acting as PD-L1 silencing compounds. The first series included about 30 compounds from which we selected a few hits with a high affinity (nM) for the recombinant PD-L1 protein and a good capacity to block the interaction between PD-L1 and PD-1 in a cellular system [14]. The phenyl-pyrazolone scaffold of the molecules was selected based on edaravone (EDA, **1**), an FDA-approved drug used to treat amyotrophic lateral sclerosis (ALS). EDA does not bind to PD-L1. It functions essentially as a scavenger of oxygen-based radicals, but it is also a reactive molecule susceptible to forming stable adducts with aromatic aldehydes [15,16]. Some of our phenyl-pyrazolone derivatives cumulate the properties of EDA as antioxidant and aldehyde-reactive molecules, in addition to their inhibitory action toward PD-L1. Indeed, we have shown recently that the phenyl-pyrazolone compounds **2** and **3** (Figure 1) combine the three desired properties: (i) a tight binding to PD-L1, (ii) a scavenging of oxygen free radicals and (iii) a covalent binding to 5-formyluracil used as a model aldehyde [17].

In an extension of this drug design program, we now report the synthesis and properties of two new phenyl-pyrazolone derivatives selected for their improved properties. We choose (i) an analogue of compound **2** with an ethyl group (**4**) instead of the methyl group on the dichlorophenyl-pyrazolone unit, and (ii) an analogue of compound **3** replacing the 2-methoxyphenyl with a 2-fluorophenyl (**5**) (Figure 1). Here we report the synthesis of the compounds, their capacity to bind to PD-L1 and to inhibit the PD-1/PD-L1 interaction, their antioxidant capacity and their aldehyde reactivity using a biologically relevant aldehyde 4-hydroxynonenal (4-HNE). The study leads to the selection of compound **5** as a new leader molecule, selected notably for its capacity to induce the dimerization of PD-L1. The study provides novel perspectives for the development of a tumor-active lead compound in the series.

## 2. Results

### 2.1. Drug Synthesis

**Figure 2:**
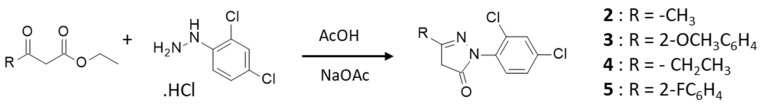
Synthetic scheme common to compounds **2**–**5**. It consists in the reaction of 2,4-dichlorophenyl-hydrazine with the indicated β-keto ester in the presence of sodium acetate, for 48 h at room temperature.

The general procedure previously described for the microwave-assisted synthesis of phenyl-pyrazolones was modified [14]. To improve the yield of the reaction and to promote cyclisation into pyrazolone, the condensation reaction between the hydrazine and the β-keto ester was realized in the presence of sodium acetate at room temperature. This reaction leading to compounds **2–5** is depicted in Figure 2. The compounds were obtained via this procedure, after purification and analytical characterization. Analytical data for compounds **3–5**, not described previously, are given in Section 4.1.

**Figure 3:**
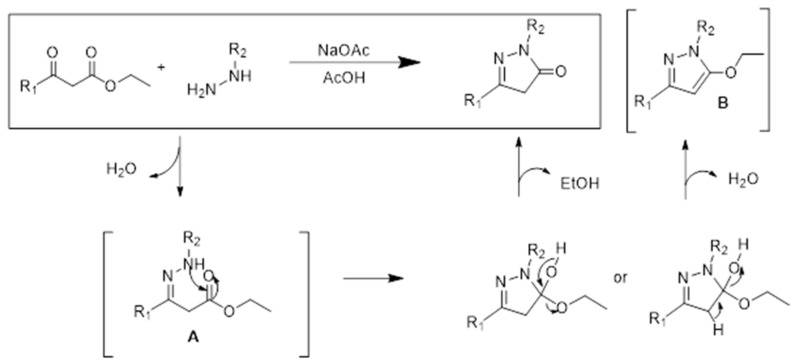
Reaction mechanism leading to the formation of the targeted pyrazolone compound together with an inactive ethoxy-pyrazole derivative. Traces of intermediates A and B can be detected by LC-MS analysis, but they were not isolated further.

It is worth noting that the yield of the reactions remains modest, in the range 30–55% depending on the compounds, due essentially to the concomitant formation of an ethoxy-pyrazole derivative (Figure 3). For one compound, the ethoxy-pyrazole derivative was isolated, purified and tested. It was totally inactive against PD-L1 in the MST assay. For the other compounds, these ethoxy-pyrazoles were not isolated. The condensation in the presence of sodium acetate at room temperature represents an improvement on the previously used microwave-assisted procedure [14], to minimize the formation of the ethoxy-pyrazole derivative.

### 2.2. Drug Binding to PD-L1 and Inhibition of PD-1 Signaling in Cells

**Figure 4:**
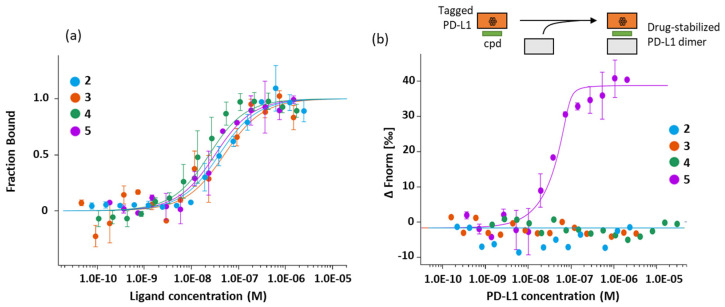
MST binding assays for PD-L1 binding: (**a**) Dose response curves for the binding of compounds **2–5** to recombinant human PD-L1. Graphs are represented as fraction bound against ligand concentration. Data represent three independent experiments (n = 3) and were fitted to a K_D_-binding model assuming a 1:1 binding stoichiometry. (**b**) Drug-induced formation of PD-L1 dimers monitored by MST. In these experiments, a fluorescent tagged PD-L1 protein incubated with the test compound is titrated with the unlabeled (non-fluorescent) protein, as represented graphically.

The ability of the phenyl-pyrazolones to interact with the PD-L1 protein was evaluated via a microscale thermophoresis (MST) assay using a recombinant human PD-L1 protein (rhPD-L1). A dose-dependent binding process was observed with each compound, providing a standard monophasic binding curve as shown in Figure 4a. From the binding plots, dissociation constants (*K*_D_) were calculated (Table 1). The four compounds exhibit a high affinity for rhPD-L1, with *K*_D_ values in the nM range. The replacement of the OCH_3_ group of **3** with an F atom reinforces the capacity of compound **5** to bind to the protein. Similarly, the methyl (**2**) to ethyl (**4**) substitution improves the binding capacity. The best binder is compound **4** with a *K*_D_ of 19 nM. For comparison, the K_D_ value measured with a human anti-PD-L1 antibody such as atezolizumab was 92 ± 11 nM. The dichlorophenyl-pyrazolone unit clearly represents a robust PD-L1 binding scaffold.

**Figure 5:**
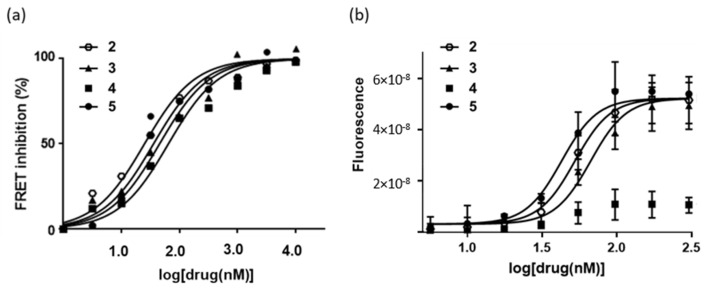
Cell-based assays: (**a**) FRET inhibition using a reporter cell-based assay. The dose-response curves were obtained upon addition of PD-L1 (10 µM) to co-transfected PD1-SHP2 cells, in the presence of increasing concentrations of the molecule under study. Data from five independent experiments (n = 5) were used to calculate IC_50_ values using the Graphpad software. (**b**) CTLL-2 cell proliferation assay. The graphic shows the dose-dependent reactivation of the proliferation of CTLL-2 cells cultured in the presence of rhPD-L1 chimera and the PD-L1 inhibitor which blocks the PD-1/PD-L1 checkpoint so as to reactivate cell proliferation. The plots were used to determine IC_50_ values with data obtained from three independent experiments (n = 3).

The cellular consequence of the PD-L1 interaction was investigated using a fluorescence resonance energy transfer (FRET) assay with the fluorescent fusion proteins SHP-2-CFP and PD-1-YFP expressed in CHO-K1 cells. Binding of PD-L1 to PD-1 triggers a downstream signaling cascade, initiated by the activation of the tyrosine phosphatase SHP-2 (Src homology region 2 domain-containing phosphatase) upon its interaction with PD-1. Therefore, a PD-L1 binding small molecule can block the FRET process between the donor (CFP) and acceptor (YFP) proteins. It is a convenient system to evidence protein–protein interactions in cellular processes and their drug regulation. The addition of rhPD-L1, which binds to cell surface PD-1, triggers a signal (fluorescence transfer) via interaction of the membrane receptor PD-1 with SHP-2. The addition of a PD-L1 binder deactivates the signal. The dichlorophenyl-pyrazolone derivatives induce a dose-dependent inhibition of the FRET signal (Figure 5a). The fitting of the dose-response curves yielded the IC_50_ values indicated in Table 1. The capacity of compound **4** to bind to PD-L1 translates into a potent inhibition of PD-L1 signaling, with IC_50_ of 12 nM, the best compound in the series for this assay. Compound **5** proved to be a little less efficient than **4** at interrupting the signalization process with an IC_50_ of 74 nM, but still efficient compared to the reference BMS-202 (Table 1). Its activity is somewhat comparable to that observed with the anti-PD-L1 atezolizumab (IC_50_ = 47 ± 11 nM) in this cell-based assay.

Next, we investigated the capacity of the compound to reactivate proliferation of CTLL-2 cells. These cytotoxic T cells expressing CD80 are maintained in a dormant state when they are cultivated in the presence of recombinant hPD-L1; their proliferation, monitored by flow-cytometry (using the CyQuant cell proliferation test), is reactivated when they are exposed to a PD-L1 inhibitor, thus mimicking a reactivation of the immune system. Surprisingly, we observed that compounds **3** and **5** only were able to stimulate proliferation of CTLL-2 cells, with IC_50_ values of 70 and 43 nM, respectively (Figure 5b). For an unknown reason, compound **4** was inactive in this assay. The replacement of the Me substituent of compound **2** with an ethyl group abolished the capacity of the product to promote CTLL-2 cell proliferation. The same result was obtained with a compound bearing a prolyl chain instead of the ethyl chain (not shown).

These observations led us to prioritize compound **5** as a hit compound owing to its potent capacity to bind to PD-L1, to interrupt PD-1/PD-L1 signaling and to reactivate CTLL-2 cell proliferation. Moreover, we selected this compound for an additional reason: its unique capacity to promote dimerization of rhPD-L1. This later property was evidenced using a specific dimerization binding assay which monitors the formation of PD-L1 dimers from a His-labeled PD-L1 protein by MST. The use of a PD-L1 protein with a polyhistidine (6xHis) tag at the C-terminus allows the non-covalent, stable, and selective labeling of the protein with a red fluorescent dye, to facilitate the formation of PD-L1 dimers. The data in Figure 4b revealed that compound **5** promoted the dimerization of PD-L1 in a dose-dependent manner, whereas compounds **3** and **4** were inactive in this assay. A *K*_D_ of 1.2 ± 0.7 nM was measured from the binding plots with compound **5**. There is no doubt that this compound is a robust inhibitor of the PD-1/PD-L1 checkpoint.

### 2.3. Antioxidant Activity

**Figure 6:**
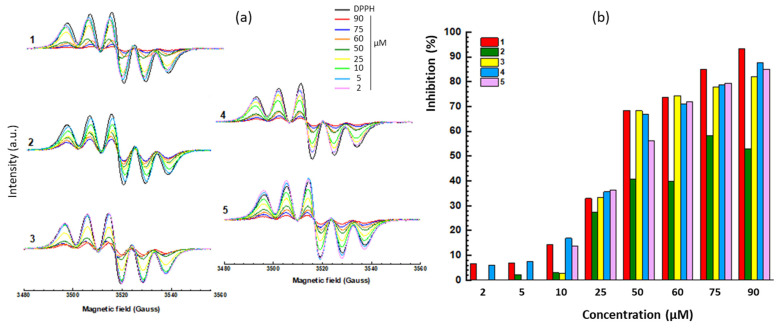
Radical scavenging: (**a**) EPR spectra of DPPH (100 µM) with increasing amounts of the indicated compound. (**b**) Comparison of the DPPH radical scavenging activity of the compounds. The DPPH concentration was 100 µM and the concentration of the test compound increased from 2 to 90 µM. The corresponding EC_50_ values are indicated in Table 2.

**Figure 7:**
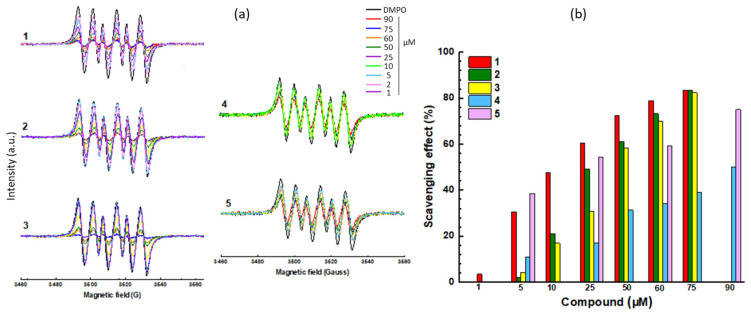
**Figure 7** Radical scavenging: (**a**) EPR spectra of the radical DMPO/^•^OCH_3_ with increasing amounts of the titled compound. (**b**) Scavenging effect (%) of the compounds. DMPO was used at 1 mM and the compound concentration varied from 1 to 75 or 90 µM. The scavenging effect (%) is defined by the ratio (area^DMPO-OMe^ − (area^Cpd^ + area^DMPO-OMe^))/area^DMPO-OMe^.

We used two electron paramagnetic resonance (EPR)-based assays to compare the antioxidant potency of the compounds. The first assay was a free radical scavenging test with 1,1-diphenyl-2-picrylhydrazyl (DPPH) to monitor the decay in the EPR signal intensity in the presence of the diamagnetic hydrazine compound. The intensity of the typical EPR spectrum of DPPH (100 μM) decreases in the presence of increasing concentration of the test compounds (Figure 6). The attenuation of the EPR signal reflects the antioxidant effect, clearly visible with each compound. From the EPR data, the concentration of the compound required to reduce the signal intensity by 50% (EC_50_) was calculated. The values determined for the two new compounds **4** and **5** were slightly reduced compared to compounds **1**–**3**, but they clearly exhibit an antioxidant effect (Table 2). The second assay relies on the use of the spin-trapping agent DMPO (5,5-dimethyl-1-pyrroline *N*-oxide) to monitor the formation of an oxygen centered radical generated under Fenton conditions (hydrogen peroxide plus ferrous ions). We used methanol as a solvent, thus leading to a monitoring of the methoxy radical DMPO/^•^OCH_3_ characterized with a typical six-line EPR signal with hyperfine coupling constants a_N_ = 13.9 G and a_H_ = 8.3 G (Figure 7). The decrease in the EPR signal was evaluated with the different compounds to compare their relative antioxidant capacity. Both compounds **4** and **5** induce a decrease in the EPR signal, but their potency is slightly reduced compared to compounds **1**–**3**. For each compound, we calculated the reaction constants (*k*_r_ in Table 2). The antioxidant capacity of compounds **4**–**5** was reduced compared to edaravone (**1**). Nevertheless, compound **5** maintains a significant activity, with a *k*_r_ value reduced by a factor 2.6 compared to the reference product **1**. The reaction rate constants are in the order of magnitude *k*_r_ = 10^11^ M^−1^ s^−1^ comparable to those measured with the conventional antioxidant compounds ascorbic acid and α-tocopherol. The value obtained with EDA is consistent with that given in the literature [18] and thus validates our measurements. The EPR measurements show that the two new derivatives maintain a marked antioxidant capacity, reduced compared to edaravone but still very significant for compounds in the phenyl-pyrazolone series.

### 2.4. Aldehyde Reactivity

**Figure 8:**
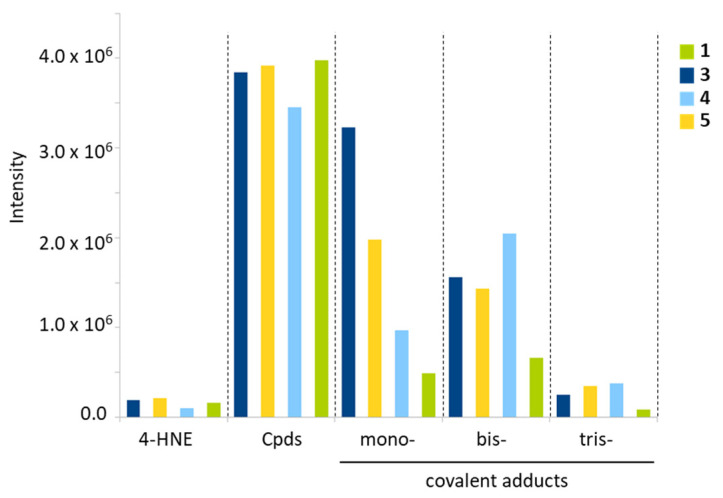
Relative proportion of the different species identified by HRMS upon reaction of 4-HNE with the indicated phenyl-pyrazolone derivatives. Columns marked “Cpds” refer to the unreacted compound. The mono-, bis-, and tris-adducts were detected by HRMS after 3 h of reaction in aqueous buffer (ammonium formate 5 mM buffer pH = 4.1 and methanol (60:40, *v*/*v*), with stirring for 180 min at 37 °C).

**Figure 9:**
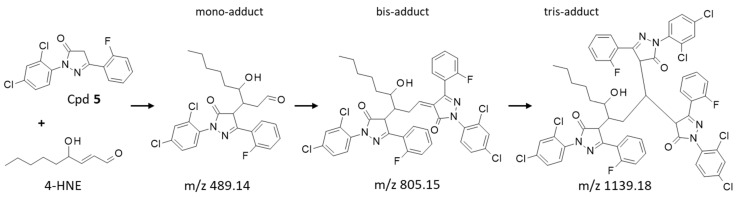
The reaction of compound **5** with 4-HNE generates three major covalent adducts: the mono-, bis- and tris-adducts, identified by their corresponding mass in the HRMS analyses. For the sake of convenience, the 3 adducts are represented in a linear form but they do not form necessarily in a sequential manner.

**Figure 10:**
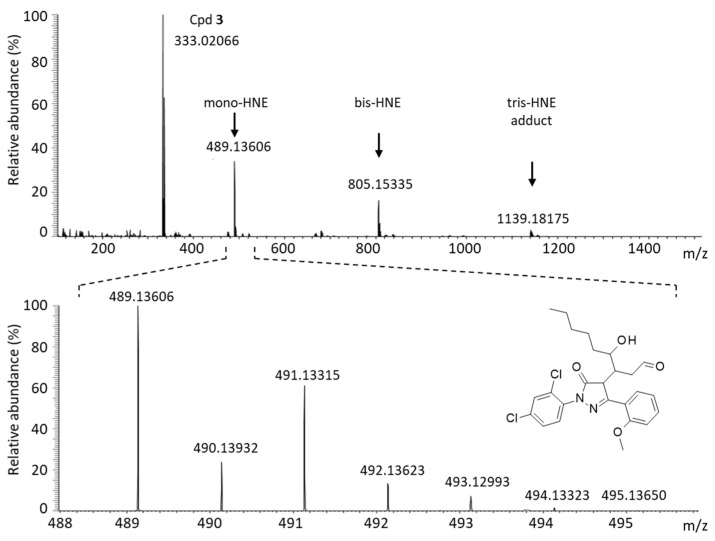
Identification of the covalent adducts formed upon reaction of compound **3** with 4-HNE by HRMS analysis. (Top spectrum) The unreacted parent product (**3**) and the different mono-, bis-, and tris-adducts are identified after 3 h of reaction in aqueous buffer (phosphate buffer 10 mM pH = 7.4, with stirring for 180 min at 37 °C). (Bottom spectrum) A detailed analysis of the 488–496 *m*/*z* range, corresponding to the mono-adduct (structure indicated).

**Figure 11:**
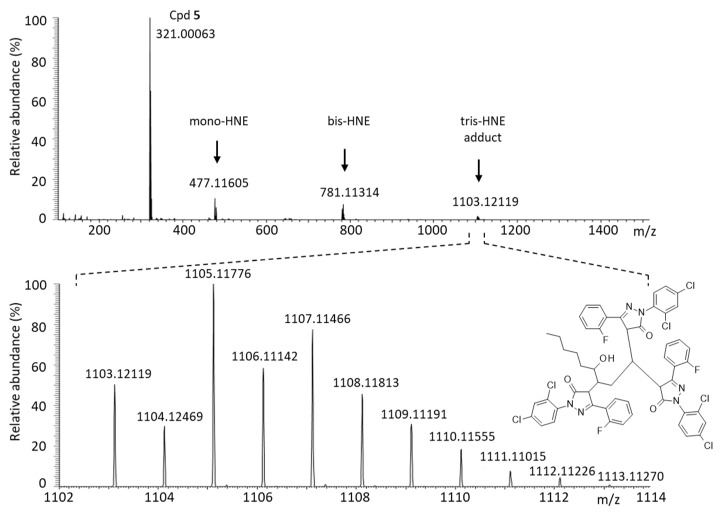
Identification of the covalent adducts formed upon reaction of compound **5** with 4-HNE by HRMS analysis. (Top spectrum) The unreacted parent product (**5**) and the different mono-, bis-, and tris-adducts are identified after 3 h of reaction in aqueous buffer (phosphate buffer 10 mM pH = 7.4, with stirring for 180 min at 37 °C). (Bottom spectrum) A detailed analysis of the 1138–1149 *m*/*z* range, corresponding to the tris-adduct (structure indicated).

Edaravone (**1**) is a reactive molecule known to form adducts with different aromatic aldehydes, such as formylpterin and vanillin [15,19]. In aqueous solution, it forms mono- and bis-adducts with the aldehyde compound vanillin [20]. The phenyl-pyrazolone derivatives can also react with aromatic aldehydes, as demonstrated previously with compounds **1**–**3** using 5-formyluracil (5fU) as a model adehyde [17]. Here we have compared the reactivity of compounds **1**–**5** using another aldehyde with a major biological relevance, 4-hydroxynonenal. 4-HNE is a product of lipid peroxidation implicated in the pathogenesis of diverse human diseases, such as diabetes, aging-related diseases and cancers [21,22]. It is an electrophilic aldehyde produced endogenously, notably inside mitochondria [23,24]. We have compared the reactivity of compounds **3**–**5** toward 4-HNE, using **1** as a control. Edaravone is known to react covalently with 4-HNE to form mono-, bis-, and tris-adducts in solution [25]. We used high-resolution mass spectrometry (HRMS) to monitor the formation of the covalent adducts generated with the phenyl-pyrazolones and 4-HNE after 3 h of incubation at physiological temperature in an aqueous buffer. The formation of mono-, bis-, and tris-adducts was detected unambiguously with each compound (Figure 8). A reaction scheme is represented in Figure 9 for compound **5**. In each case, the pyrazolone compound (nucleophile) easily reacted with 4-HNE to generate the different species, each characterized by their corresponding mass (*m*/*z*), the ring-double-bond value (rdb, valence values of elements in structure), the signal intensity and the correspondence with the theoretical *m*/*z* value (Δppm) (Table 3). Typical mass spectra obtained with compounds **3** and **5** in the presence of 4-HNE are shown in Figure 10 and Figure 11. The different species can be easily identified, with the isotopic pattern characteristic for the presence of chlorine and carbon atoms (chlorine and carbon cluster isotopes). In all cases, the species formed were identified without any ambiguity from their relative abundances. For example, the isotopic pattern of the monoadduct from compound **3** and 4-HNE is characterized by 6 signals and an isotopic distribution of 100% (^12^C and ^35^Cl), 25% (one ^13^C and ^35^Cl), 63% (^12^C and ^35^Cl and ^37^Cl), 16% (one ^13^C and ^35^Cl and ^37^Cl), 10% (^12^C and two ^37^Cl) and 3% (one ^13^C and two ^37^Cl). These values were in accordance with the theorical abundances, calculated from the exactive software for the isotopic pattern of the ions from *m*/*z* = 489.13606 to *m*/*z* = 494.13323. Table 3 summarizes the data obtained with each compound and each species.

Unsurprisingly, edaravone (**1**) was found to be extremely reactive toward 4-HNE, in agreement with its known capacity to restrict accumulation of 4-HNE in cells [25]. It is a suppressor of 4-HNE-associated oxidative stress after hemorrhage [26]. In the phenyl-pyrazolone series, the most efficient 4-HNE-binder is compound **3**, followed by compounds **4** and **5**. These two later molecules behaved slightly differently. Compound **4** essentially formed bis-adducts after 180 min of reaction, whereas compound **5** gave more mono- than bis-adducts. However, overall, the two compounds can be considered as 4-HNE-reactive molecules. A reaction scheme for the formation of the different adducts with compound **5** is indicated in Figure 9. The three types of adducts are represented, but the predominant form identified is the mono-adduct which is formed from the Michael addition of the nucleophile. In all cases in our series, the nucleophile additions are not observed to the aldehyde carbon of 4-HNE as previously described for edaravone [25]. Overall, the data confirm the reactivity of this class of aryl-pyrazolone toward aldehydes and point out the capacity of the products to function as 4-HNE blocking agents.

## 3. Discussion

A variety of small molecules targeting PD-L1 and inhibiting the PD-1/PD-L1 checkpoint have been designed over the past few years. The structural diversity is relatively large, including biphenyls, phtalimides, triazines, quinazolines, indolines, and others [4,27,28,29,30,31,32,33]. There are also a few molecules targeting the receptor PD-1, not the ligand PD-L1 [11,34]. The field is very active and novel tumor-active small molecules targeting PD-L1 are regularly discovered. For example, a recent study underlined the potency of a novel N-biphenyl phtalimide derivative (S4-1) to trigger T-cell activation via a mechanism implicating drug-induced dimerization of PD-L1 [35]. Regularly, promising results have been reported at the experimental level. However, thus far, only a few compounds have been advanced to human clinical trials, with mitigated results in most cases. Efforts continue to identify more potent compounds.

One potential option to reinforce the antitumor potency of PD-L1-targeted small molecules is to confer additional properties, contributing to the anticancer action. Inhibiting the PD-1/PD-L1 checkpoint is important, but often not sufficient to insure an efficient antitumor action. In this frame, we considered the development of the PyrDLones, which are pyrazolone derivatives targeting PD-L1 endowed with marked antioxidant properties and a capacity to react with aldehydes. These molecules present an aryl-pyrazolone scaffold reminiscent to the core structure of the antioxidant drug edaravone (**1**), used to treat amyotrophic lateral sclerosis (ALS) and acute ischemic stroke [36,37]. Edaravone is a potent antioxidant and an aldehyde-reactive compound [16]. The implication of an oxidative stress in cancer is largely recognized. It is commonly associated with the development and progression of solid tumors, such as liver and skin cancers [38,39]. Antioxidant compounds can be useful to alleviate the oxidative stress and also to reduce the oxidative damages associated with chemotherapy. Fortifying the antioxidant defense is a strategy considered to reduce side effects of chemotherapy [40]. The antioxidant properties of the phenyl-pyrazolones can contribute to the anticancer activity of the molecules and facilitate drug combination. An anticancer activity has been observed in a murine syngeneic model of colon cancer with another derivative (unpublished data).

Similarly, we consider that the aldehyde reactivity of the molecules could be a positive element to reduce damages caused by peroxidation metabolites such as 4-HNE which strongly impact mitochondria function and dynamics [24]. 4-HNE is strongly associated with inflammation and with several diseases such as pulmonary fibrosis, diabetes and cancer [21,41]. It is considered as a second messenger of free radicals acting as signaling molecules and as cytotoxic products of lipid peroxidation, via covalent modification of macromolecules [42]. Beyond 4-HNE, aldehydes are considered as carcinogenic molecules and their implication of health damages is well documented [43,44,45]. It makes sense to design PD-L1 inhibitors endowed with antioxidant properties. There is an increasing interest in small-molecule immuno-oncology drugs, notably for molecules with immune-modulatory effects that can synergize with the action of immune checkpoint inhibition antibodies [1].

From the work reported here, we selected compound **5** (and its derivatives) as a new candidate for in vivo studies in a xenograft model. This compound presents improved properties compared to the previous candidate **3**, notably a capacity to induce the formation of PD-L1 dimers (Figure 4b). This is a key attribute for small molecule inhibitors targeting PD-L1 [46]. Dimerization PD-L1 favors its reduction from the cell surface (internalization of the receptor) and its lysosomal degradation [47,48,49]. Compound **5** presents improvements compared to compound **3** in terms of PD-L1 inhibition, while maintaining a marked antioxidant capacity and a reactivity toward toxic aldehydes. Its solubility and microsomal stability are also slightly improved. The stability (t_1/2_) measured in the presence of mouse liver microsomes was 479.7 and 317 µg/min/mg for **3** and **5**, respectively. The stability remains insufficient, but it is similar to that of the drug edaravone (t_1/2_ = 317 µg/min/mg under strictly identical conditions). The limited metabolic stability of the pyrazolones is a key point in the series. We are currently testing a novel series to improve this aspect, via new substitution of the pyrazole ring. There are options to improve the bioavailability of these compounds using specific formulations, as it has been done with edaravone [50,51]. There are also options to improve the bioavailability by chemical modification of the compound while preserving its key attributes. New compounds derived from **5** will be reported in our forthcoming study.

## 4. Materials and Methods

### 4.1. Synthesis of the Compounds

Materials. Chemical reactions were analyzed by analytical thin-layer chromatography (TLC) on 0.2 mm, Polygram SIL G/UV254 plates (Macherey-Nagel); compounds were visualized by UV (254 and 366 nm). Silica gel Kieselgel Si 60, 0.015–0.040 mm (Macherey-Nagel) was used to perform flash chromatography. Melting points (Mp) were obtained with a capillary apparatus (Büchi 535) and were uncorrected. Nuclear magnetic resonance (^1^H and ^13^C NMR) spectra were recorded at room temperature on a Bruker AC 300 spectrometer. Tetramethylsilane (TMS) was used as an internal standard and CDCl_3_ or DMSO-*d*_6_ as the solvents. ^1^H NMR analyses were obtained at 300 MHz (s: singlet, d: doublet, t: triplet, q: quadruplet, quint, quintuplet, sext.: sextuplet, hept.: heptuplet, dd: double doublet, m: multiplet); whereas ^13^C NMR analyses were obtained at 75.4 MHz. The chemical shifts (δ) are given in parts per million (ppm) relative to TMS (δ = 0.00). All compounds were analyzed by Acquity UPLC I Class coupled to QDa mass spectrometer (Performance Waters), column C18 1.7 microns, eluted by a gradient H_2_O/CH_3_CN/5 mM ammonium formate, pH 3.8. The detection mode is Full Scan ESI+/−. IR spectra were recorded on a Bruker Vector 22 FT-IR spectrometer. The purity of all compounds was determined by HPLC using a Chromazing column 4.6 × 150 mm, 5 μm, 100 Å, mobile phase: CH_3_CN/H_2_O/HCOOH: Sol A (800/200/0.1) or Sol B (700/300/0.1). WATERS 600 pump chromatograph equipped with a WATERS 2487 dual absorption wavelength UV detector (λ = 254 nm and 366 nm). Retention time was obtained with flow rates of 1 mL/min. The acquisition time is 10 or 20 min.

Synthetic procedure. To a solution of β-ketoester (1 eq, 1 mmol) in AcOH (5 mL) were added sodium acetate (0.5 eq) and hydrazine hydrochloride (1 eq). The reaction mixture was stirred for 48 h at room temperature. After reaction, the solvent was evaporated under reduced pressure. The residue was extracted with ethyl acetate (10 mL), washed with brine (10 mL). The organic layer was dried over MgSO_4_ and evaporated under reduced pressure.

*1-(2,4-Dichlorophenyl)-3-(2-methoxyphenyl)-1H-pyrazol-5(4H)-one* (**3**): The oil obtained was purified by chromatography on silica gel with cyclohexane/ethyl acetate (7:3) as eluent and recrystallized with ethanol. White solid. Yield: 51%. mp: 188 °C. Rf (cyclohexane/ethyl acetate 1:1): 0.6. ^1^H NMR (DMSO): 3.86 (s, 3H); 6.02 (s, 1H); 6.95 (t, 1H, *J* = 7.4 Hz,); 7.08 (d, 1H, *J* = 8.2 Hz); 7.29 (t, 1H, *J* = 7.2 Hz); 7.56 (d, 2H, *J* = 0.8 Hz); 7.81 (dd, 1H, *J* = 7.9 Hz, *J*’ = 1.4 Hz); 7.84 (s, 1H); 11,35 (s, 1H). LC-MS (ESI^+^) *m*/*z* 335.0 (MH^+^); tr = 2.92 min. ^13^C NMR (DMSO) δ 157, 154, 148, 135, 134, 133, 132, 130, 129, 128, 127, 122, 121, 112, 88, 56.
*1-(2,4-dichlorophenyl)-3-ethyl-1H-pyrazol-5(4H)-one* (**4**): Compound was purified by flash chromatography using with cyclohexane/ethyl acetate (1:1). Orange solid. Yield 29%. mp: 160 °C. Rf (cyclohexane/ethyl acetate 1:1): 0.3. ^1^H NMR (CDCl_3_) δ 7.51–7.50 (d, *J* = 2.0 Hz, 1H); 7.37–7.34 (d, *J* = 9.2 Hz, 1H); 7.32–7.29 (Dd, *J* = 9.2 Hz, *J^’^* = 2.0 Hz, 1H); 3.39 (s, 2H); 2.53–2.46 (d, *J* = 7.7 Hz, 1H); 1.25–1.20 (t, *J* = 7.7 Hz, 3H). LC-MS (ESI) *m*/*z* 258 (MH^+^), t_r_ 2.36 min. ^13^C NMR (CDCl_3_) δ 171, 161, 135, 133, 132, 130, 129, 128, 40, 25, 11.
*1-(2,4-Dichlorophenyl)-3-(2-fluorophenyl)-1H-pyrazol-5-ol* (**5**): The product was recrystallized with methanol. White solid. Yield: 34%. mp: 229 °C. Rf (cyclohexane/ethyl acetate 7:3): 0.7. ^1^H NMR (DMSO): 5.93 (d, 1H, *J* = 4,2 Hz); 7.21–7.39 (m, 3H); 7,60 (m, 2H); 7.88–7.94 (m, 2H); 11.65 (s, 1H). LC-MS (ESI^+^) *m*/*z* 323.3 (MH^+^); tr = 2.97 min. ^13^C NMR (DMSO) δ 161, 158, 155, 146, 135, 134, 132, 131, 130, 129, 128, 125, 121, 116, 87.

The metabolic stability of the compounds in mice liver microsomes was investigated using liver microsomes from male mice (BD Gentest™) with propranolol as a positive control, as described previously [52]. Mouse plasma CD-1 was used to investigate the plasma stability of the molecules, with enalapril as a positive control, as described recently [14].

### 4.2. Microscale Thermophoresis (MST)

The MST procedure used to quantify inhibition of PD-L1 binding to PD-1 has been described [53,54]. Briefly, MST was conducted using a NT.115 Pico MST instrument (Nano Temper Technologies GmbH) equipped with red and blue filters. Recombinant Human PD-L1 (rhPD-L1) His-tag protein (R&D Systems, reference #9049-B7-100), diluted to 200 nM in PBS-T buffer (supplied by the vendor), was labeled with Monolith His-Tag Labeling Kit RED-tris-NTA (Nano Temper). The RED-tris-NTA dye was diluted in PBS-T to 100 nM. The mix was incubated in the dark for 30 min at room temperature. Ligands were diluted with a serial 1:1 ratio of 16 gradients. Then the labeled protein and ligands were mix with 1:1 ratio and incubated in the dark for 15 min at room temperature. Capillaries were filled individually and loaded into the instrument. Data, acquired with high MST power and 100% LED, were analyzed using the MO Control Software from Nano Temper. To generate the dose-response curves, ligand-dependent changes in MST are plotted as Fnorm (‰) values vs. ligand concentration.

### 4.3. MST Dimerization Binding Assay

A labeled His-PD-L1 protein was titrated against an unlabeled His-PD-L1 protein with or without compounds of interest. The test compound (1 µM) and the His-labeled PD-L1 protein (10 nM) are kept constant whereas the unlabeled His-PD-L1 (1 µM) varies with a serial 1:1 ratio of 16 gradients. MST was performed using the same instrument as mentioned above. His-PD-L1 protein (Biotechne # 9049-B7-100) was diluted to 200 nM in commercial PBS-T buffer and was labeled as indicated above. The RED-tris-NTA dye was diluted in PBS-T to 100 nM. The mix was incubated at room temperature in the dark for 30 min. Capillaries were filled individually.

### 4.4. Fluorescence Resonance Energy Transfer (FRET) Assay

A cellular model was developed using CHO-K1 cells overexpressing of the protein of interest, PD-1. The model involves the cellular expression of a PD-1-YFP fluorescent protein (fluorescent emission of acceptor Yellow Fluorescent Protein (λ_Ex_ = 514 nm λ_Em_ = 527 nm) and a second fluorescent protein SHP-2 tagged with CFP (donor Cyan Fluorescent Protein, λ_Ex_ = 433 nm, λ_Em_ = 475 nm). CFP and YFP are variants of the green fluorescent protein (GFP) from *Aequorea victori* [55]. Src homology 2 (SHP-2) is a tyrosine phosphatase selectively recruited by PD-1 to initiate T cell inactivation [56]. To measure the FRET signal, 10,000 cells co-transfected with the PD1-SHP2 vector and activated by PD-L1 addition (10 µM) were used. A Spectramax i3 (Molecular Devices^®^, San Jose, CA, USA, Country) was used for the FRET measurements, configured according to the selected fluorochromes with their excitation and emission spectra. Endpoint reading was performed at the center of the well. The reading time is 2 min for a 96-well plate. The drug was tested at various concentrations (typically from 0.5 to 400 mM) and IC_50_ values were calculated by curve fitting using the GraphPad Prism v8 software.

### 4.5. Cell Proliferation Assay

The CyQUANT cell proliferation kit (Thermofischer, Waltham, MA, USA) was used to evaluate the growth of the CTLL-2 cell line (ATCC). Cells were prepared by growing them in the presence or absence of rhPD-L1 chimera (R&D) plus the test compound, and then transferring them to microplates for assay according to manufacturer’s instructions (Molecular Probes C35011). A cell-based standard curve was first established, with the amount of fluorescence correlated to the quantity of cells in the wells. Cells were incubated with increasing concentrations of compounds, over a period 72 h. Positive (nivolumab) and negative (no rhPD-L1 Fc chimera) controls were performed to calibrate the assay. IC_50_ were determined for the restoration of proliferation by global curve fitting with Graphpad. Experiments were repeated three times and IC_50_ values averaged.

### 4.6. EPR Experiments

An X-band Brüker Elexsys E500 spectrometer operating at 9.86 GHz was used to perform CW-EPR experiments. CW-EPR spectra were recorded at room temperature with a microwave power of 5.024–10.02 mW and a modulation amplitude of **1–2** G that considers non-saturation conditions. The free radical scavenging molecule DPPH (1,1-diphenyl-2-picrylhydrazyl) and spin trap agent DMPO (5,5-dimethyl-1-pyrroline *N*-oxide) were used to monitor the antioxidant activity of the test compounds. The solution mixture (50 μL) was filled into a glass capillary, placed in 4 mm quartz EPR tubes for analysis. The inhibition of DPPH (%) was calculated with the equation: I (%) = ((I_0_ − I)/I_0_) × 100 where I_0_ is the area of the EPR spectrum of DPPH (control sample), and I the area of the DPPH spectrum with antioxidant compound. The rate constant for the reaction of antioxidant compounds with ^•^OCH_3_ is expressed by the equation: 1/R_a_ = 1/R_f_ + ((K_r_ [AH])/(k_a_ [DMPO] R_f_)) where R_f_, and R_a_ indicate EPR signal area (double integration of EPR signal) of ^•^OCH_3_, DMPO/^•^OCH_3_, respectively [18,57]. *k*_a_ and [DMPO] are constants, and a plot of 1/*R*_a_ vs. [AH] (concentration of antioxidant compound) gives a straight line with 1/*R*_f_ as an intercept and *k*_r_/(*k*_a_[DMPO]*R*_f_) as the slope. The rate constant for the reaction of ^•^OCH_3_ with antioxidant compounds (*k*_r_) is expressed by the equation: *k*_r_ = (*k*_a_ [DMPO] × slope)/− intercept.

### 4.7. Aldehyde (4-HNE) Reactivity Measurements

High resolution mass spectrometry (HRMS) and spectra were recorded with an Exactive Plus mass spectrometer (Thermo Fisher Scientific, San Jose, CA, USA) equipped with a heated electrospray ionisation probe (HESI -II). Each mass analyzer was calibrated with Pierce^®^ESI positive and negative ion calibration solutions each week (Thermo Fisher Scientific). Optimization of voltages, gas values, and temperatures applied for ion transfer and ionization was performed in negative mode. The tuning parameters were optimized separately with reaction mixture obtained from reaction of nucleophile with the aldehyde. This was completed by infusing first individual solutions of each nucleophile or the aldehyde at 10 pmol/µL. Each reaction mixture was performed during 180 min at 37 °C with the compound at 0.5 mM in phosphate buffer 10 mM, pH = 7.4 and then the reaction mixture was diluted 1/400 in MeOH. The solution was injected with a syringe at a flow rate of 10 µL/min. MS parameters were: sheath gas flow rate 45; auxiliary gas flow rate 10 arbitrary units (nitrogen was used as auxiliary and sheath gas); spray voltage −3.50 kV; capillary temperature 320 °C; auxiliary gas heater temperature 320 °C and S-lens RF level 50. The ions between *m*/*z* 150–1500 were scanned in high-resolution mode of the instrument. The identification of the reaction products was performed by HRMS data as elementary analysis parameters (*m*/*z* values, relative abundances of isotopic pattern, Ring Double Bond values and mass accuracies lower than 5 ppm).

## 5. Conclusions

The work reported here points out the capacity of small molecules including a dichlorophenyl-pyrazole core to function as inhibitors of the PD-1/PD-L1 immune checkpoint. The leading compound **5** binds to PD-L1 and induces its dimerization so as to block the downstream signaling activity. In parallel, the molecules display marked antioxidant effects via the scavenging of oxygen-based radicals. They can also form covalent adducts with the lipid peroxidation product 4-hydroxynonenal (4-HNE), considered a toxic aldehyde. This is a further step toward the design of antitumor PyrDLOnes.

## Figures and Tables

**Table 1:** PD-L1 binding and regulatory properties of the test compounds.

Compounds	PD-L1 Binding (K_D_, nM) ^a^	Bioactivity (IC_50_, nM) ^b^	Proliferation (IC_50_, nM) ^c^
**2**	34 ± 3	23 ± 3	53 ± 11
**3**	45 ± 7	44 ± 9	79 ± 7
**4**	19 ± 3	12 ± 5	*nd*
**5**	27 ± 4	74 ± 7	43 ± 9
BMS-202	*nd*	124 ± 12	53 ± 17

^a^ Affinity for PD-L1 measured by microscale thermophoresis (MST). ^b^ Capacity of the compound to block the interaction between PD-L1 and PD-1 in cells, analyzed with a fluorescence resonance energy transfer (FRET) assay. ^c^ Ability of the compound to reactivate CTLL-2 cell proliferation. *nd* = not determined.

**Table 2:** Antioxidant properties of the test compounds.

Compounds	DMPO		DPPH EC_50_ (μM)
	*k*_r_ (10^10^ M^−1^ s^−1^)	*k*_r_/*k*_a_	
**1**	25.9	60	35.81
**2**	19.3	45	36.46
**3**	18.8	44	34.02
**4**	4.40	10	40.94
**5**	9.74	23	38.18

DMPO was used at 1 mM. *k*_r_/*k*_a_ is the ratio of the reaction rate constant (*k*_r_) with the test compounds and the reaction rate constant for DMPO (k_a_ (DMPO-^•^OH) = 4.3 × 10^9^ M^−1^ s^−1^).

**Table 3:** HRMS analysis of the reaction of the compounds with 4-hydroxynonenal (4-HNE).

Products [M–H]^-^	Formula	Ion *m*/*z* Values (Observed)	Intensities	Errors (Δppm)	rdb
Cpd **1** (edaravone) - 4-HNE - EDA - mono-adduct - bis-adduct - tris-adduct	C_9_H_15_O_2_ C_10_H_9_N_2_O_2_ C_19_H_25_N_2_O_3_ C_29_H_33_N_4_O_3_ C_39_H_43_N_6_O_4_	155.10692 173.0712 329.18719 485.25602 659.33535	1.63 × 10^5^ 3.98 × 10^6^ 4.88 × 10^5^ 6.60 × 10^5^ 8.37 × 10^4^	1.8 1.5 1.7 2.7	2.5 7.5 8.5 15.5 21.5
Cpd **3** - 4-HNE - Cpd **3** - mono-adduct - bis-adduct - tris-adduct	C_9_H_15_O_2_ C_16_H_11_N_2_O_2_Cl_2_ C_25_H_27_N_2_O_4_Cl_2_ C_41_H_37_N_4_O_5_Cl_4_ C_57_H_49_N_6_O_7_Cl_6_	155.10692 333.02066 489.13606 805.15335 1139.18175	1.92 × 10^5^ 3.84 × 10^6^ 3.23 × 10^6^ 1.56 × 10^6^ 2.53 × 10^5^	1.6 4.0 3.7 2.6 2.5	2.5 11.5 12.5 23.5 33.5
Cpd **4** - 4-HNE - Cpd **4** - mono-adduct - bis-adduct - tris-adduct	C_9_H_15_O_2_ C_11_H_9_N_2_OCl_2_ C_20_H_25_N_2_O_3_Cl_2_ C_31_H_33_N_4_O_3_Cl_4_ C_42_H_43_N_6_O_4_Cl_6_	155.10689 255.00989 411.12532 649.13249 905.14908	1.01 × 10^5^ 3.45 × 10^6^ 9.70 × 10^5^ 2.05 × 10^6^ 3.76 × 10^5^	1.5 4.9 4.0 3.6 2.1	2.5 7.5 8.5 15.5 21.5
Cpd **5** - 4-HNE - Cpd **5** - mono-adduct - bis-adduct - tris-adduct	C_9_H_15_O_2_ C_15_H_8_N_2_OCl_2_F C_24_H_24_N_2_O_3_Cl_2_F C_39_H_31_N_4_O_3_Cl_4_F_2_ C_54_H_40_N_6_O_4_Cl_6_F_2_	155.10691 321.00062 477.11605 781.11314 1103.12119	2.11 × 10^5^ 3.92 × 10^6^ 1.98 × 10^6^ 1.43 × 10^6^ 3.51 × 10^5^	1.3 4.5 3.6 2.8 2.9	2.5 11.5 12.5 23.5 33.5

RDB, Ring Double Bound value. Δppm, delta ppm from the theoretical *m*/*z* values (negative mode).

## Data Availability

Not applicable.

## Sample Availability

Samples of compounds 2-5 are available from the authors.
